# Association of Computed Tomography Measures of Muscle and Adipose Tissue and Progressive Changes throughout Treatment with Clinical Endpoints in Patients with Advanced Lung Cancer Treated with Immune Checkpoint Inhibitors

**DOI:** 10.3390/cancers15051382

**Published:** 2023-02-22

**Authors:** Azim Khan, Christopher J. Welman, Afaf Abed, Susan O’Hanlon, Andrew Redfern, Sara Azim, Pedro Lopez, Favil Singh, Adnan Khattak

**Affiliations:** 1 Northam Regional Hospital, Northam, WA 6401, Australia; 2 Department of Medical Imaging, Fiona Stanley Hospital, Perth, WA 6150, Australia; 3 Peel Health Campus, Mandurah, WA 6210, Australia; 4 Department of Medical Oncology, Fiona Stanley Hospital, Murdoch, WA 6150, Australia; 5 School of Medicine and Pharmacology, UWA, Perth, WA 6009, Australia; 6 Fiona Stanley Hospital, Murdoch, WA 6150, Australia; 7 Exercise Medicine Research Institute, Edith Cowan University, Joondalup, WA 6027, Australia; 8 School of Medical and Health Sciences, Edith Cowan University, Joondalup, WA 6027, Australia; 9 Pleural Medicine Unit, Institute for Respiratory Health, Perth, WA 6009, Australia

**Keywords:** immunotherapy, cancer, muscle mass, fat mass, overall survival

## Abstract

**Simple Summary:**

The impact of sarcopenia (i.e., progressive and generalised loss of skeletal muscle mass) and obesity on survival are substantially investigated in cancer patients. However, the relationship between sarcopenia and mortality is quite unclear in patients with lung cancer treated with immunotherapy, while the prognostic value of obesity remains controversial. These issues are potentially related to the obesity paradox and lack of precise measures of body composition on survival. As a result, we aimed to explore the associations between measures of skeletal muscle mass and adiposity (i.e., intramuscular, visceral and subcutaneous adipose tissue) and changes during treatment with disease progression and overall survival in patients with advanced lung cancer receiving immunotherapy. Our results demonstrated that rather than sarcopenia, higher intramuscular and subcutaneous adipose tissue are associated with better prognosis during immunotherapy. These findings are of great importance for clinical practice and may inform specific and tailored therapies to improve immunotherapy prognosis.

**Abstract:**

To investigate the association between skeletal muscle mass and adiposity measures with disease-free progression (DFS) and overall survival (OS) in patients with advanced lung cancer receiving immunotherapy, we retrospectively analysed 97 patients (age: 67.5 ± 10.2 years) with lung cancer who were treated with immunotherapy between March 2014 and June 2019. From computed tomography scans, we assessed the radiological measures of skeletal muscle mass, and intramuscular, subcutaneous and visceral adipose tissue at the third lumbar vertebra. Patients were divided into two groups based on specific or median values at baseline and changes throughout treatment. A total number of 96 patients (99.0%) had disease progression (median of 11.3 months) and died (median of 15.4 months) during follow-up. Increases of 10% in intramuscular adipose tissue were significantly associated with DFS (HR: 0.60, 95% CI: 0.38 to 0.95) and OS (HR: 0.60, 95% CI: 0.37 to 0.95), while increases of 10% in subcutaneous adipose tissue were associated with DFS (HR: 0.59, 95% CI: 0.36 to 0.95). These results indicate that, although muscle mass and visceral adipose tissue were not associated with DFS or OS, changes in intramuscular and subcutaneous adipose tissue can predict immunotherapy clinical outcomes in patients with advanced lung cancer.

## 1. Introduction 

In recent years, immune checkpoint inhibitors (ICIs) or immunotherapies, such as nivolumab, pembrolizumab and ipilimumab, have evolved rapidly in medical oncology. The utilisation of ICIs has become a key component for managing a variety of malignancies including lung cancer, resulting in an unprecedented survival advantage over standard therapies such as radiation therapy and chemotherapy. While chemotherapy acts directly on cancer cells inhibiting the cell cycle, ICIs are antibodies targeting the programmed death 1 (PD-1), programmed death-ligand (PD-L1) or cytotoxic T-lymphocyte-associated protein 4 (CTLA-4), blocking key regulatory signals that dampen immune responses in the tumour microenvironment. As a result, ICIs counteract immune suppression allowing for tumour reactive T cells to mount an antitumour response utilising the patient’s immune system to target the malignancy [1]. These therapies have shown promising effects in the treatment of lung cancer, as well as a selection of other solid tumours and haematologic malignancies [2,3,4,5].

Several studies have pointed out a significant relationship between immunotherapy and multiple variables on overall survival. Among potential factors, sarcopenia (i.e., progressive and generalised loss of skeletal muscle mass [6]) has emerged as an important prognostic factor in different groups of cancer patients [7]. However, the relationship between sarcopenia and overall survival in patients treated with immunotherapy is still unclear [8,9]. While studies present a significant association between sarcopenia and shorter overall survival [8], others have no significant relationship [9]. For example, in a previous study with small-cell lung cancer patients receiving salvage anti-PD-1 immunotherapy (n = 105), patients presenting with low levels of muscle mass (i.e., sarcopenic patients) had a ~200% greater risk of all-cause mortality compared to those with higher levels of muscle mass [8]. In contrast, there was no difference in overall survival between sarcopenic and non-sarcopenic patients with solid metastatic tumours treated with ICIs (n = 261) [9]. Moreover, the prognostic value of obesity in various malignancies is unknown and remains controversial for the survival of various malignancies [10]. Although previous studies indicated a potential association between body mass index (BMI) and overall survival in advanced cancer patients treated with immunotherapy [11,12], others have demonstrated no significant association between BMI and clinical endpoints [13]. These conflicting results, potentially related to the obesity paradox (i.e., inconsistency concerning the role of obesity on survival), preclude us from understanding the role of fat mass components (i.e., visceral adipose tissue or subcutaneous adipose tissue) on survival in this population [14,15]. For example, while visceral adipose tissue (VAT) secretes various cytokines and cytokine-like factors, which potentially enhance cancer progression [16,17], derived factors from the subcutaneous adipose tissue can increase insulin sensitivity and lipid metabolism potentially resulting in an improved survival [18]. Therefore, although BMI is a much simpler and widely used tool in clinical practice, it does not reflect individual components of body weight such as fat distribution or muscle quantity and quality. 

As a result, this study aims to investigate the associations between measures of skeletal muscle mass, intramuscular adipose tissue, subcutaneous adipose tissue, visceral adipose tissue and visceral-to-subcutaneous adipose tissue index and changes throughout treatment with disease progression and overall survival in patients with advanced lung cancer receiving immunotherapy.

## 2. Materials and Methods

### 2.1. Study Population

Retrospective analyses of computerised tomography (CT) imaging and electronic medical record data were performed for all patients treated with immunotherapy who presented to Fiona Stanley Hospital, Western Australia between March 2014 and June 2019. A total of 124 patients with lung cancer were identified on immunotherapy. Patients without CT imaging data were excluded from the final cohort, resulting in a total of 97 patients included for further analyses. Demographic, pathological and survival information were obtained via electronic medical record review. The duration of follow-up was 60 months from the first presentation to the date of death for deceased patients or the date of last documented encounter for surviving patients. Demographic and clinical data such as sex, age, BMI, smoking habits, Eastern Cooperative Oncology Group (ECOG) performance status (PS), distant metastases, cancer type, treatment regimens, progression-free survival (PFS) and overall survival (OS) were collected by self-report and medical records, respectively. Our study was approved by the Hospital Ethics Committee (RGS0000003289) and conducted in compliance with the Helsinki Declaration.

### 2.2. Assessment of Muscle Mass and Fat Mass Parameters

CT scans were at a median of 20 [interquartile range (IQR): 8 to 31] days before commencing immunotherapy treatment. CT scans of the abdomen/pelvis were performed as part of recommended staging pathway and retrieved from the hospital imaging PACS/RIS system (version 6.7.0.6011; Agfa, Mortsel, Belgium). A single 3 mm axial slice through the middle of the L3 vertebral body was retrieved using the sagittal reformatted images with the morphologic L5/S1 junction as reference. These images were imported into SliceOmatic (version 5.0 Rev 12; TomoVision, Magog, QC, Canada) and analysed using the ABACS mode (version 6 Rev-7b; Voronoi Health Analytics, Coquitlam, BC, Canada). If there was an artifact at this level, the nearest artifact-free contiguous slice above or below this level was utilised. A visual colour-coded overlay was reviewed to assess for correct segmentation; any errors were manually corrected using *Edit mode* and following standard anatomic boundaries. Area measurements (cm^2^) were obtained by auto-segmentation using the default Hounsfield unit (HU) thresholds and skeletal muscle was determined in the range of −29 to 150 HU, including the skeletal muscle compartment of psoas, paraspinal and abdominal wall musculature. Intramuscular adipose tissue (IMAT) was determined in the range of −190 to −30 HU, visceral adipose tissue (VAT) in the range of −150 to −50 HU and subcutaneous adipose tissue (SAT) in the range of −190 to −30 HU. Visceral-to-subcutaneous adipose tissue ratio was defined as the ratio between VAT and SAT values. Values were normalised to height squared (m^2^) to derive skeletal muscle, IMAT, VAT, SAT and VAT/SAT indexes. 

For further analysis, the skeletal muscle index was analysed as a categorical variable with two levels corresponding to sarcopenia (skeletal muscle index < 43 cm^2^·m^−2^ and BMI < 25 kg·m^−2^, or skeletal muscle index < 53 cm^2^·m^−2^ and BMI ≥ 25 kg·m^−2^) and non-sarcopenia (skeletal muscle index ≥ 43 cm^2^·m^−2^ and BMI ≥ 25 kg·m^−2^, or skeletal muscle index ≥ 53 cm^2^·m^−2^ and BMI < 25 kg·m^−2^), as previously established [19]. Considering the lack of cut-off values for adiposity measures, median values based on our sample were used to categorise patients with higher and lower levels of IMAT, VAT, SAT and VAT/SAT indexes. Relative changes (%) were calculated as $\frac{index_{follow-up}}{index_{baseline}}*100\%$, with a threshold of 10% utilised to categorise groups with the lowest and highest index changes throughout treatment.

### 2.3. Assessment of Outcomes

The primary outcome was overall survival, defined as deaths as a result of any cause, while disease progression defined as an increase in the size of the tumour by 20% was secondary. Vital causes and causes of death were obtained via electronic medical record review. Follow-up time for overall mortality was calculated as the time from CT scans to death from any cause or the end of follow-up (i.e., 60 months following the time of the first scan).

### 2.4. Statistical Analyses

Analyses were performed using SPSS v.27 (Armonk, IBM Corp., NY, USA) and R Core Team (2013). Differences in overall mortality between groups based on sarcopenia, IMAT, SAT, VAT and VAT/SAT variables were assessed using the Kaplan–Meier method and the log-rank test. Paired-sample *t*-test was used to compare values between the first and second CT scans during immunotherapy. The hazard ratios (HRs) for the associations of skeletal muscle index, IMAT, SAT, VAT and VAT/SAT ratio indexes with overall mortality and disease progression were estimated in separate models using Cox proportional hazards regression. Logistic regression was used to determine the impact of body composition components on the occurrence of adverse events ≥ grade 2. Odds ratios (ORs) and 95% CIs were reported. Models were adjusted for age, BMI, cancer type and stage. A *p*-value of ≤0.05 was considered statistically significant and point estimates were presented with 95% confidence interval. 

## 3. Results 

### 3.1. Patient Characteristics

Patient characteristics are presented in Table 1. Patients were 67.5 ± 10.2 years of age (mean ± standard deviation) with a BMI of 26.1 ± 4.9 kg·m^−2^. Most patients were overweight/obese (60.8%). The majority of patients had adenocarcinoma (62.9%), followed by squamous cell carcinoma (29.9%). Most patients were treated with second line immunotherapy (75.3%). A total of 81 patients were stage IV (84.4%) and had metastatic disease present in more than two sites (22.9%), bone (17.1%), lymph node (8.6%), liver (5.7%), adrenal (2.9%) and brain (2.9%). In this cohort, the most common immunotherapy agent was Nivolumab (58.8%), followed by Pembrolizumab (24.7%) and Atezolumab (16.5%). A total number of 96 patients had disease progression and died during follow-up (99.0%), with median disease progression of 11.3 (IQR: 4.9 to 20.4) months and 15.4 (IQR: 7.2 to 24.0) months, respectively.

### 3.2. Association of Body Composition Components with Disease Progression and Overall Survival

**Figure 1:**
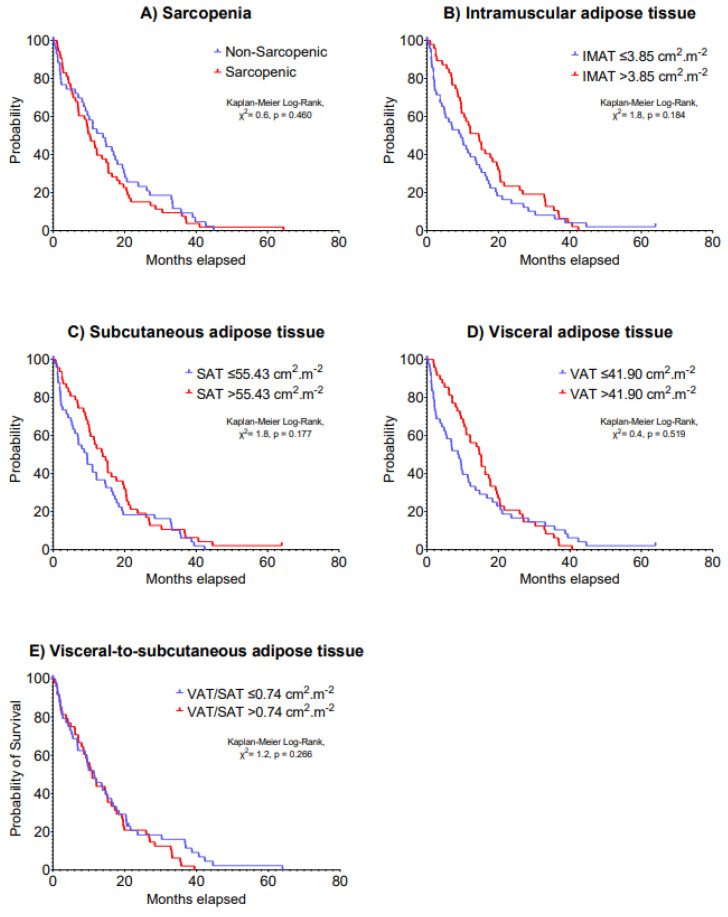
Kaplan–Meier curves of 5-year disease progression according to (**A**) sarcopenia, (**B**) intramuscular adipose tissue, (**C**) subcutaneous adipose tissue, (**D**) visceral adipose tissue and (**E**) visceral-to-subcutaneous adipose tissue at baseline in patients with advanced lung cancer undergoing immunotherapy.

**Figure 2:**
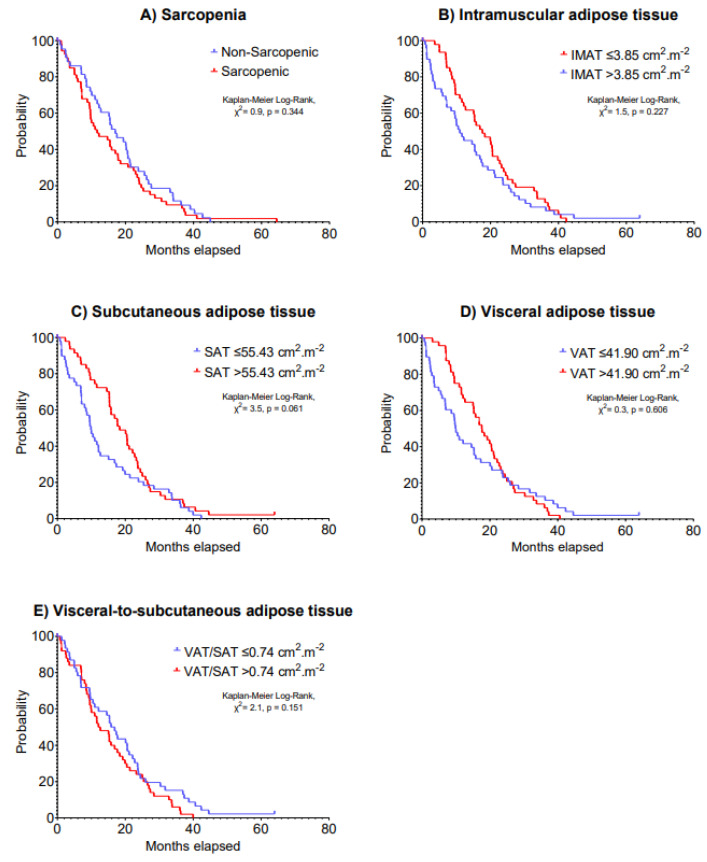
Kaplan–Meier curves of 5-year overall survival according to (**A**) sarcopenia, (**B**) intramuscular adipose tissue, (**C**) subcutaneous adipose tissue, (**D**) visceral adipose tissue and (**E**) visceral-to-subcutaneous adipose tissue at baseline in patients with advanced lung cancer undergoing immunotherapy.

The median IMAT, SAT, VAT and VAT/SAT ratio index values were 3.85, 55.43, 41.90 and 0.74 cm^2^·m^−2^, respectively. Multivariable models indicated no significant associations of sarcopenia, IMAT, SAT, VAT and VAT/SAT ratio indexes at baseline with 5-year disease progression (HR: 0.69–1.25, *p* = 0.199–0.877) and 5-year overall survival (HR: 0.69–1.34, *p* = 0.123–0.724) in patients with advanced lung cancer undergoing immunotherapy (Table 2). Kaplan–Meier analyses stratifying patients according to body composition components cut-off values on 5-year disease progression and overall survival are presented in Figure 1 and Figure 2, respectively (*p* = 0.061–0.606).

**Figure 3:**
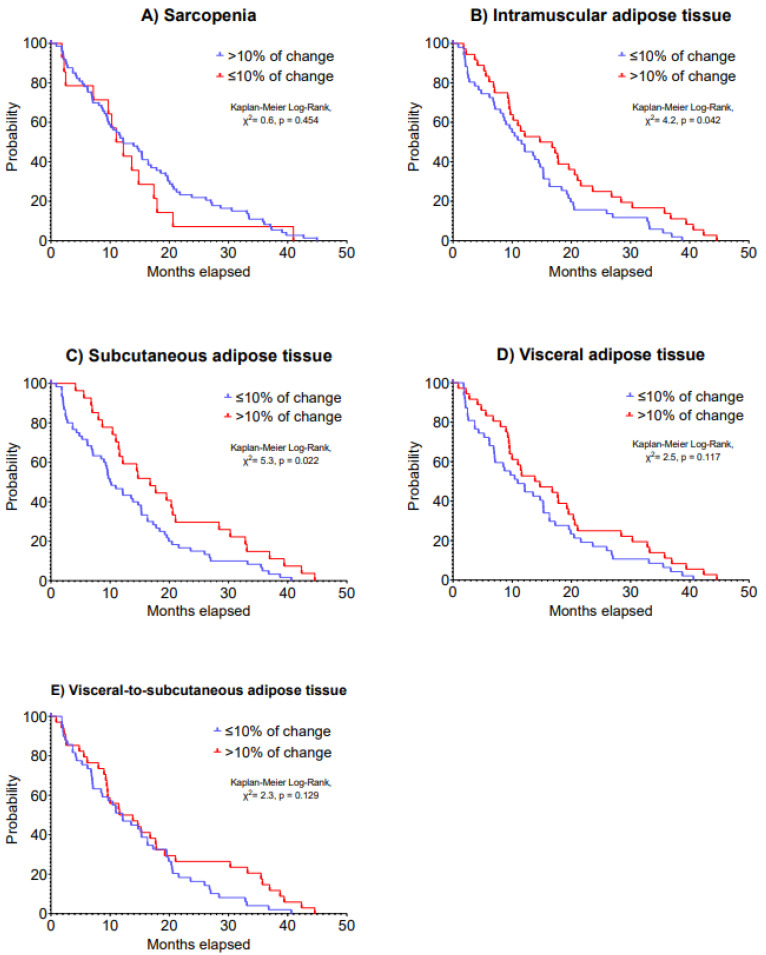
Kaplan–Meier curves of 5-year disease progression according to changes in (**A**) sarcopenia, (**B**) intramuscular adipose tissue, (**C**) subcutaneous adipose tissue, (**D**) visceral adipose tissue and (**E**) visceral-to-subcutaneous adipose tissue in patients with advanced lung cancer undergoing immunotherapy.

A second CT scan was performed in 88 patients as presented in Table 3. Changes in skeletal muscle, IMAT, SAT, VAT and VAT/SAT ratio indexes were not statistically significant following a median time of 15.4 months after the first CT scan (IQR: 7.1 to 26.5 days). Although changes in sarcopenia, VAT and VAT/SAT ratio indexes were not associated with 5-year disease progression (HR: 0.63–1.24, *p* = 0.064–0.484), >10% increases in IMAT (HR: 0.60, 95% CI: 0.38 to 0.95) and SAT indexes (HR: 0.59, 95% CI: 0.36 to 0.95) were associated with improved 5-year disease progression (*p* = 0.028 and 0.029; Table 4). Patients with a >10% increase in IMAT index presented a median disease progression of 15.9 (IQR: 8.8 to 24.6) months vs. 11.7 (IQR: 5.5 to 19.0) months in patients with a ≤10% decrease in IMAT index (Kaplan–Meier Log-Rank, χ^2^ = 4.2, *p* = 0.042). Likewise, patients who had a >10% increase in SAT index presented a median disease progression of 16.9 (IQR: 10.8 to 29.6) months vs. 10.2 (IQR: 4.7 to 18.8) months of patients who had a decrease in SAT index (Kaplan–Meier Log-Rank, χ^2^ = 5.3, *p* = 0.022). Kaplan–Meier analysis on 5-year disease progression is presented in Figure 3. 

**Figure 4:**
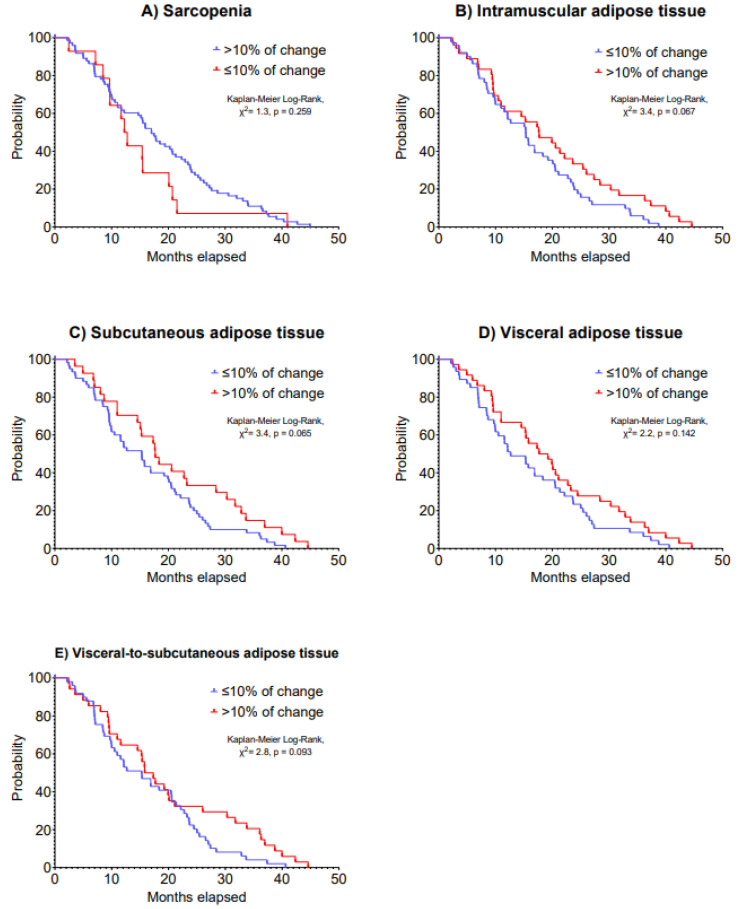
Kaplan–Meier curves of 5-year overall survival according to changes in (**A**) sarcopenia, (**B**) intramuscular adipose tissue, (**C**) subcutaneous adipose tissue, (**D**) visceral adipose tissue and (**E**) visceral-to-subcutaneous adipose tissue in patients with advanced lung cancer undergoing immunotherapy.

Regarding overall survival, a >10% increase in IMAT was associated with improved 5-year overall survival (HR: 0.60, 95% CI: 0.37 to 0.95, *p* = 0.031; Table 5). Patients who had an increase of 10% in IMAT presented a median overall survival of 17.8 (IQR: 9.6 to 27.9) months vs. 15.5 (IQR: 8.5 to 23.2) months of patients who had a decrease in this outcome (Kaplan–Meier Log-Rank, χ^2^ = 3.4, *p* = 0.067). Kaplan–Meier analysis on 5-year overall survival is presented in Figure 4.

### 3.3. Association of Body Composition Components with Immune-Related Adverse Events

Thirty-six adverse events (43.4%) were observed during immunotherapy. Of these, a total of 11 grade 2 (13.3%) and 5 grade 3 events (6.0%) were observed. No associations were observed between sarcopenia, IMAT, SAT, VAT and VAT/SAT ratio indexes with high-grade adverse events during immunotherapy (OR: 0.95–2.00, *p* = 0.279–0.947).

## 4. Discussion

The present study reported the associations between radiological measures of muscle and adipose tissue with disease progression and overall survival in patients with advanced lung cancer receiving immunotherapy. The main findings were: (i) muscle mass index at the time of or during immunotherapy was not associated with disease progression or overall survival; and (ii) patients with lung cancer presenting with increases of 10% in intramuscular and subcutaneous adipose tissue following treatment were at a ~40% decreased risk of disease progression and overall survival compared to those presenting with lower levels, regardless of age, BMI, cancer type and stage.

The significant association of sarcopenia with poor disease prognosis has been observed in several papers across different types of cancer [20,21,22]. Interestingly, the majority of studies reporting such findings in the field of immunotherapy were undertaken in patients with lung cancer [8,20,23,24,25,26]. As far as we know, this is one of the few studies [25] undertaken in patients with lung cancer mainly with adenocarcinoma and squamous cell carcinoma (~93% of the sample). Our study indicates that sarcopenia is not significantly associated with disease progression or overall survival in this population with advanced cancer receiving immunotherapy. As observed in our results, the presence of sarcopenia at the start of immunotherapy or a reduction of 10% in skeletal muscle mass index were not associated with disease progression and mortality. However, this result disagrees with previous studies undertaken in patients mainly with non-squamous lung cancer [8,26], which indicate that tumour histology may affect the interaction between sarcopenia and immunotherapy in patients with advanced lung cancer. Nevertheless, lower levels of muscle mass may still affect other important components of immunotherapy such as inflammation, cachexia and physical disability. Consequently, more research is required to elucidate the importance of sarcopenia for other important clinical measures.

The investigation of obesity in immunotherapy is challenging given the confounding factors associated with the obesity paradox [15] and its role in cancer dynamics [27,28]. We observed that intramuscular and subcutaneous adipose tissue could be a predictive marker for improved survival when increased throughout the treatment course. The subcutaneous adipose tissue derives a range of factors such as leptin that could act to improve insulin sensitivity and lipid metabolism [17,18,29]. As a result, this could potentially increase overall survival in this group of patients and represent an important measure during the cancer survivorship. However, the result that an increase in intramuscular adipose tissue could improve survival was unexpected. While previous studies identified a significant association of intramuscular adipose tissue with shorter survival in women with non-metastatic breast cancer [30] and men with hormone-sensitive prostate cancer [31], others did not observe a significant association in metastatic breast cancer [32] or advanced non-small-cell lung cancer treated with immunotherapy [33,34]. Moreover, previous studies have demonstrated that increased intramuscular fat is related to increased frailty and sarcopenia [35] and impaired physical function [36]. In addition, others indicate that increased intramuscular fat is associated with poor survival and increased risk of hospitalisation in older adults or critically ill patients [37,38]. Therefore, the interaction between intramuscular fat and immunotherapy is yet to be determined in this setting.

Interestingly, we also observed an unexpectedly longer 5-year disease progression compared to other large immunotherapy randomised controlled studies [39,40,41,42]. While we observed a median disease progression time of 11.3 months, a range of 3.0 to 5.0 months was reported in these trials [39,40,41,42]. The reasons are likely multifactorial and related to our smaller sample size and retrospective nature compared to these large randomised controlled trials [39,40,41,42]. Additionally, we observed high PDL1% values in our sample (median of 60%) and this may also account to a long disease progression as PDL1% is associated with improved survival even when using monotherapy agents in advanced non-small-cell lung cancer. Other factors such as mixed cancer stages (~16% stage III) and treatment line (~25% first treatment line) are different than these previous immunotherapy trials [39,40,41,42] and may affect disease progression. Our cohort also presented more favourable histology (i.e., adenocarcinoma) and tumour burden may be different as 40% did not present distant metastasis. These factors may play a role in disease progression.

Some limitations are worthy of comment. The retrospective nature of the study and the heterogeneity of CT scans may limit our ability to extrapolate our findings to a large scale. Future studies should undertake prospective models to assess the influence of body composition changes on clinical endpoints, as well as reporting the time of body composition assessment. In addition, the lack of standardisation (i.e., cut-off values), and this is due to variability in underlying technique without clear standardisation, makes comparison difficult to assess radiological measures of muscle and adipose tissue and affects our ability to provide more meaningful recommendations based on our findings. Although the use of body composition is promising, critical and technical studies are required to understand the relationship of sarcopenia with clinical endpoints and to inform specific and tailored interventions in patients treated with immunotherapy. Finally, we could not estimate the impact of sarcopenic obesity in our sample. This is an emergent topic in oncology given the high risk of mortality and severe complications experienced by patients during systemic and surgical cancer treatments. Future studies are required to investigate the impact of sarcopenic obesity in lung cancer patients during immunotherapy and identify clinical management strategies for this population.

## 5. Conclusions

In conclusion, our findings are that rather than muscle mass and visceral adipose tissue, changes in intramuscular and subcutaneous adipose tissue can predict immunotherapy clinical outcomes regardless of age, BMI, cancer type and stage. This result provides new insights into the assessment of body composition in patients with advanced lung cancer undergoing immunotherapy. Consequently, future research should seek to assess a larger sample size of patients undergoing immunotherapy to further elucidate the influence of body composition, specifically monitoring intramuscular and subcutaneous adipose tissues.

## Figures and Tables

**Table 1:** Characteristics of patients with lung cancer.

Characteristics	Patients (n = 97)
Age, mean ± SD, years	67.5 ± 10.2
Males, n (%)	55 (56.7%)
Height, mean ± SD, cm	168.1 ± 8.7
Weight, mean ± SD, kg	73.8 ± 15.6
BMI, mean ± SD, kg·m^−2^	26.1 ± 4.9
BMI, categories, n (%)	
Underweight	4 (4.1%)
Normal weight	34 (35.1%)
Overweight	43 (44.3%)
Obese	16 (16.5%)
Current smoker, n (%) ^a^	74 (83.1%)
Tumour type, n (%)	
Adenocarcinoma	61 (62.9%)
Adeno-squamous	1 (1.0%)
Large cell carcinoma	2 (2.1%)
Poorly differentiated	3 (3.1%)
Pleomorphic	1 (1.0%)
Squamous cell carcinoma	29 (29.9%)
Cancer stage, n (%) ^a^	
III	15 (15.6%)
IV	81 (84.4%)
Metastasis location, n (%) ^a^	
No metastasis	14 (40%)
Adrenal	1 (2.9%)
Bone	6 (17.1%)
Brain	1 (2.9%)
Liver	2 (5.7%)
Lymph node	3 (8.6%)
≥2 sites	8 (22.9%)
Treatment line, n (%)	
First line treatment	24 (24.7%)
Second line treatment	73 (75.3%)
Treatment, n (%)	
Atezolumab	16 (16.5%)
Nivolumab	57 (58.8%)
Pembrolizumab	24 (24.7%)
ECOG, n (%) ^a^	
0	49 (51.0%)
1	34 (35.5%)
2	12 (12.5%)
3	1 (1.0%)
Neutrophils, mean ± SD	6.6 ± 4.4
Lymphocytes, mean ± SD	1.5 ± 0.8
Haemoglobin, mean ± SD	125.2 ± 20.4
Platelets, mean ± SD	306.1 ± 122.0
Neutrophils-to-lymphocytes ratio, mean ± SD	6.9 ± 11.6
PD-L1 levels, median (IQR), %	60.0 (1.0 to 80.0)

BMI, Body mass index; ECOG, Eastern Cooperative Oncology Group performance status; IQR, interquartile range; SD, standard deviation; ^a^, missing data: current smoker, n = 8; cancer stage, n = 1; TNM, n = 16; metastasis location, n = 62; ECOG, n = 1.

**Table 2:** Hazard ratios and 95% confidence intervals for associations between measures of skeletal muscle, intramuscular adipose tissue, subcutaneous adipose tissue, visceral adipose tissue and visceral-to-subcutaneous adipose tissue indexes and 5-year risk of disease progression and overall survival.

Variables	N	5-Year Disease Progression		5-Year Overall Survival	
		HR (95% CI)	*p*-Value	HR (95% CI)	*p*-Value
Sarcopenia					
Non-sarcopenic	44	Reference		Reference	
Sarcopenic	53	1.22 (0.81 to 1.85)	0.338	1.29 (0.85 to 1.95)	0.227
Intramuscular adipose tissue index					
≤3.85 cm^2^·m^−2^	49	Reference		Reference	
>3.85 cm^2^·m^−2^	48	0.83 (0.51 to 1.33)	0.430	0.88 (0.54 to 1.42)	0.594
Subcutaneous adipose tissue index					
≤55.43 cm^2^·m^−2^	49	Reference		Reference	
>55.43 cm^2^·m^−2^	48	0.69 (0.43 to 1.10)	0.199	0.69 (0.43 to 1.10)	0.123
Visceral adipose tissue index					
≤41.90 cm^2^·m^−2^	49	Reference		Reference	
>41.90 cm^2^·m^−2^	48	0.96 (0.58 to 1.60)	0.877	1.10 (0.66 to 1.83)	0.724
Visceral-to-Subcutaneous adipose tissue index					
≤0.74 cm^2^·m^−2^	48	Reference		Reference	
>0.74 cm^2^·m^−2^	49	1.25 (0.82 to 1.90)	0.305	1.34 (0.88 to 2.04)	0.178

All models were adjusted for age, BMI, cancer type and cancer stage. 95% CI, 95% confidence intervals; HR, hazard ratio.

**Table 3:** Changes in skeletal muscle, intramuscular adipose tissue, subcutaneous adipose tissue, visceral adipose tissue and visceral-to-subcutaneous adipose tissue indexes during immunotherapy.

Outcomes	n	Baseline	Post-Assessment	Mean Difference
		Mean ± SD	Mean ± SD	Mean ± SD (95% CI)
Skeletal muscle index, cm^2^·m^−2^	88	43.8 ± 8.5	43.7 ± 9.4	−0.1 ± 6.6 (−1.5 to 1.3)
Intramuscular adipose tissue index, cm^2^·m^−2^	88	4.5 ± 2.3	4.7 ± 2.2	0.2 ± 1.3 (−0.1 to 0.5)
Subcutaneous adipose tissue index, cm^2^·m^−2^	88	64.2 ± 36.5	65.5 ± 38.6	1.3 ± 17.5 (−2.4 to 5.0)
Visceral adipose tissue index, cm^2^·m^−2^	88	52.5 ± 34.7	54.6 ± 36.8	2.1 ± 18.9 (−2.0 to 6.2)
Visceral-to-subcutaneous adipose tissue ratio, cm^2^·m^−2^	88	1.0 ± 0.7	1.0 ± 0.7	0.0 ± 0.3 (−0.1 to 0.1)

**Table 4:** Hazard ratios and 95% confidence intervals for associations between changes in measures of skeletal muscle, intramuscular adipose tissue, subcutaneous adipose tissue, visceral adipose tissue and visceral-to-subcutaneous adipose tissue indexes and 5-year risk of disease progression and overall survival.

Variables	N	5-Year Disease Progression		5-Year Overall Survival	
		HR (95% CI)	*p*-Value	HR (95% CI)	*p*-Value
Sarcopenia					
>10% of change	74	Reference		Reference	
≤10% of change	14	1.24 (0.68 to 2.23)	0.484	1.39 (0.77 to 2.52)	0.277
Intramuscular adipose tissue index					
≤10% of change	52	Reference		Reference	
>10% of change	36	0.60 (0.38 to 0.95)	0.028	0.60 (0.37 to 0.95)	0.031
Subcutaneous adipose tissue index					
≤10% of change	61	Reference		Reference	
>10% of change	27	0.59 (0.36 to 0.95)	0.029	0.64 (0.39 to 1.03)	0.066
Visceral adipose tissue index					
≤10% of change	48	Reference		Reference	
>10% of change	36	0.66 (0.42 to 1.04)	0.075	0.67 (0.43 to 1.07)	0.093
Visceral-to-Subcutaneous adipose tissue index					
≤10% of change	50	Reference		Reference	
>10% of change	34	0.63 (0.39 to 1.03)	0.064	0.61 (0.38 to 0.99)	0.045

All models were adjusted for age, BMI, cancer type and cancer stage.

**Table 5:** Odds ratio and 95% confidence intervals for associations between measures of skeletal muscle, intramuscular adipose tissue, subcutaneous adipose tissue, visceral adipose tissue and visceral-to-subcutaneous adipose tissue indexes and risk of treatment toxicity.

Variables	N	Treatment Toxicity		
		OR	95% CI	*p*-Value
Sarcopenia				
Non-sarcopenic	44	Reference		
Sarcopenic	53	2.00	0.59 to 7.66	0.279
Intramuscular adipose tissue index				
≤3.85	49	Reference		
>3.85	48	0.95	0.21 to 4.22	0.947
Subcutaneous adipose tissue index				
≤55.43	49	Reference		
>55.43	48	1.23	0.28 to 5.48	0.782
Visceral adipose tissue index				
≤41.90	49	Reference		
>41.90	48	1.35	0.30 to 6.50	0.696
Visceral-to-Subcutaneous adipose tissue index				
≤0.74	48	Reference		
>0.74	49	1.57	0.44 to 5.95	0.490

All models were adjusted for age, BMI, cancer type and cancer stage.

## Data Availability

The data used in the present study will be made available upon request to the corresponding author.
